# Evaluating the Capabilities of Generative AI Tools in Understanding Medical Papers: Qualitative Study

**DOI:** 10.2196/59258

**Published:** 2024-09-04

**Authors:** Seyma Handan Akyon, Fatih Cagatay Akyon, Ahmet Sefa Camyar, Fatih Hızlı, Talha Sari, Şamil Hızlı

**Affiliations:** 1 Golpazari Family Health Center Bilecik Turkey; 2 SafeVideo AI San Francisco, CA United States; 3 Graduate School of Informatics Middle East Technical University Ankara Turkey; 4 Department of Internal Medicine Ankara Etlik City Hospital Ankara Turkey; 5 Faculty of Medicine Ankara Yildirim Beyazit University Ankara Turkey; 6 Department of Computer Science Istanbul Technical University Istanbul Turkey; 7 Department of Pediatric Gastroenterology, Children Hospital, Ankara Bilkent City Hospital Ankara Yildirim Beyazit University Ankara Turkey

**Keywords:** large language models, LLM, LLMs, ChatGPT, artificial intelligence, AI, natural language processing, medicine, health care, GPT, machine learning, language model, language models, generative, research paper, research papers, scientific research, answer, answers, response, responses, comprehension, STROBE, Strengthening the Reporting of Observational Studies in Epidemiology

## Abstract

**Background:**

Reading medical papers is a challenging and time-consuming task for doctors, especially when the papers are long and complex. A tool that can help doctors efficiently process and understand medical papers is needed.

**Objective:**

This study aims to critically assess and compare the comprehension capabilities of large language models (LLMs) in accurately and efficiently understanding medical research papers using the STROBE (Strengthening the Reporting of Observational Studies in Epidemiology) checklist, which provides a standardized framework for evaluating key elements of observational study.

**Methods:**

The study is a methodological type of research. The study aims to evaluate the understanding capabilities of new generative artificial intelligence tools in medical papers. A novel benchmark pipeline processed 50 medical research papers from PubMed, comparing the answers of 6 LLMs (GPT-3.5-Turbo, GPT-4-0613, GPT-4-1106, PaLM 2, Claude v1, and Gemini Pro) to the benchmark established by expert medical professors. Fifteen questions, derived from the STROBE checklist, assessed LLMs’ understanding of different sections of a research paper.

**Results:**

LLMs exhibited varying performance, with GPT-3.5-Turbo achieving the highest percentage of correct answers (n=3916, 66.9%), followed by GPT-4-1106 (n=3837, 65.6%), PaLM 2 (n=3632, 62.1%), Claude v1 (n=2887, 58.3%), Gemini Pro (n=2878, 49.2%), and GPT-4-0613 (n=2580, 44.1%). Statistical analysis revealed statistically significant differences between LLMs (*P*<.001), with older models showing inconsistent performance compared to newer versions. LLMs showcased distinct performances for each question across different parts of a scholarly paper—with certain models like PaLM 2 and GPT-3.5 showing remarkable versatility and depth in understanding.

**Conclusions:**

This study is the first to evaluate the performance of different LLMs in understanding medical papers using the retrieval augmented generation method. The findings highlight the potential of LLMs to enhance medical research by improving efficiency and facilitating evidence-based decision-making. Further research is needed to address limitations such as the influence of question formats, potential biases, and the rapid evolution of LLM models.

## Introduction

Artificial intelligence (AI) has revolutionized numerous fields, including health care, with its potential to enhance patient outcomes, increase efficiency, and reduce costs [1]. AI devices are divided into 2 main categories. One category uses machine learning techniques to analyze structured data for medical applications, while the other category uses natural language processing methods to extract information from unstructured data, such as clinical notes, thereby improving the analysis of structured medical data [2]. A key development within natural language processing has been the emergence of large language models (LLMs), which are advanced systems trained on vast amounts of text data to generate human-like language and perform a variety of language-based tasks [3]. While deep learning models recognize patterns in data [4], LLMs are trained to predict the probability of a word sequence based on the context. By training on large amounts of text data, LLMs can generate new and plausible sequences of words that the mode has not previously observed [4]. ChatGPT, an advanced conversational AI technology developed by OpenAI in late 2022, is a general-purpose LLM [5]. GPT is part of a growing landscape of conversational AI products, with other notable examples including Llama (Meta), Jurassic (Ai21), Claude (Anthropic), Command (Cohere), Gemini (formerly known as Bard), PaLM, and Bard (Google) [5]. The potential of AI systems to enhance medical care and health outcomes is highly promising [6]. Therefore, it is essential to ensure that the creation of AI systems in health care adheres to the principles of trust and explainability. Evaluating the medical knowledge of AI systems compared to that of expert clinicians is a vital initial step to assess these qualities [5,7,8].

Reading medical papers is a challenging and time-consuming task for doctors, especially when the papers are long and complex. This poses a significant barrier to efficient knowledge acquisition and evidence-based decision-making in health care. There is a need for a tool that can help doctors to process and understand medical papers more efficiently and accurately. Although LLMs are promising in evaluating patients, diagnosis, and treatment processes [9], studies on reading academic papers are limited. LLMs can be directly questioned and can generate answers from their own memory [10,11]. This has been extensively studied in many papers. However, these pose the problem of artificial hallucinations, which are inaccurate outputs, in LLMs. The retrieval augmented generation (RAG) method, which intuitively addresses the knowledge gap by conditioning language models on relevant documents retrieved from an external knowledge source, can be used to overcome this issue [12].

The STROBE (Strengthening the Reporting of Observational Studies in Epidemiology) checklist provides a standardized framework for evaluating key elements of observational study and sufficient information for critical evaluation. These guidelines consist of 22 items that authors should adhere to before submitting their manuscripts for publication [13-15]. This study aims to address this gap by evaluating the comprehension capabilities of LLMs in accurately and efficiently understanding medical research papers. We use the STROBE checklist to assess LLMs’ ability to understand different sections of research papers. This study uses a novel benchmark pipeline that can process PubMed papers regardless of their length using various generative AI tools. This research will provide critical insights into the strengths and weaknesses of different LLMs in enhancing medical research paper comprehension. To overcome the problem of “artificial hallucinations,” we implement the RAG method. RAG involves providing the LLMs with a prompt that instructs them to answer while staying relevant to the given document, ensuring responses align with the provided information. The results of this study will provide valuable information for medical professionals, researchers, and developers seeking to leverage the potential of LLMs for improving medical literature comprehension and ultimately enhance patient care and research efficiency.

## Methods

### Design of Study

This study uses a methodological research design to evaluate the comprehension capabilities of generative AI tools using the STROBE checklist.

### Paper Selection

**Figure 1:**
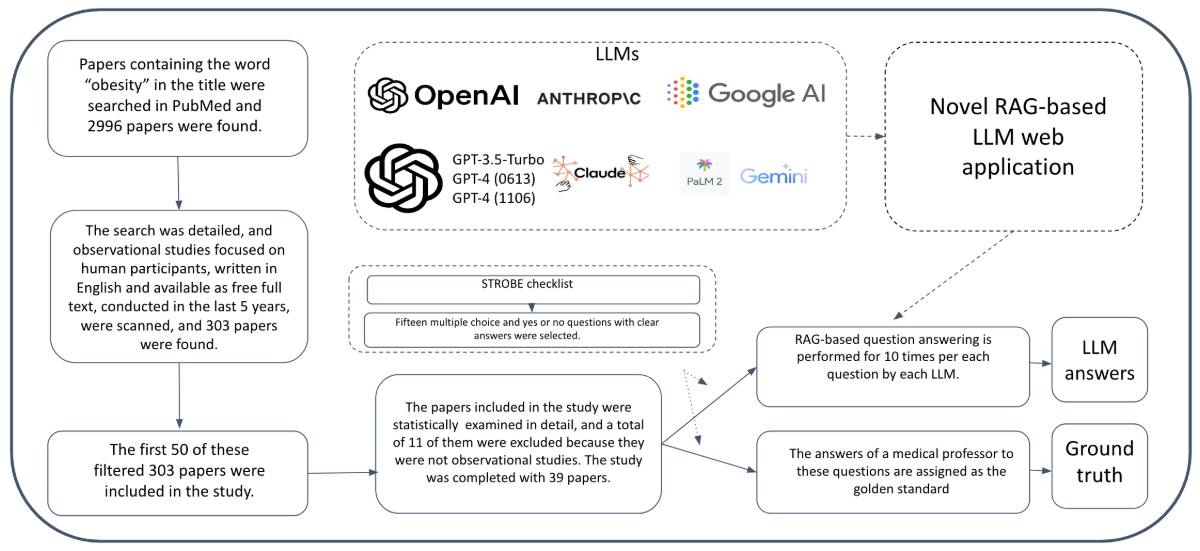
Flowchart: recruitment and data collection process for evaluating LLM comprehension of medical research papers. LLM: large language model; RAG: retrieval augmented generation.

We included the first 50 observational studies conducted within the past 5 years that were retrieved through an advanced search on PubMed on December 19, 2023, using “obesity” in the title as the search term. The included studies were limited to those written in English, available as free full text, and focusing specifically on human participants (Figure 1). The papers included in the study were statistically examined in detail, and a total of 11 of them were excluded because they were not observational studies. The study was completed with 39 papers. A post hoc power analysis was conducted to assess the statistical power of our study based on the total correct responses across all repetitions. The analysis excluded GPT-4-1106 and GPT-3.5-Turbo-1106 due to their similar performance and the significant differences observed between other models. The power analysis, conducted using G*Power (version 3.1.9.7; Heinrich-Heine-Universität Düsseldorf), indicated that all analyses exceeded 95% power. Thus, the study was completed with the 39 selected papers, ensuring sufficient statistical power to detect meaningful differences in LLM performance.

### Benchmark Development

This study used a novel benchmark pipeline to evaluate the understanding capabilities of LLMs when processing medical research papers. To establish a reference standard for evaluating the LLMs’ comprehension, we relied on the expertise of an experienced medical professor and an epidemiology expert doctor. The professor, with their extensive medical knowledge, was tasked with answering 15 questions derived from the STROBE checklist, designed to assess key elements of observational studies and cover different sections of a research paper (Table 1). The epidemiology expert doctor, with their specialized knowledge in statistical analysis and epidemiological methods, provided verification and validation of the professor’s answers, ensuring the rigor of the benchmark. The combined expertise of both professionals provided a robust and reliable reference standard against which the LLMs’ responses were compared.

**Table 1:** The questions derived from the STROBE (Strengthening the Reporting of Observational Studies in Epidemiology) checklist for observational study and answers.

Questions		Answers
**Title and abstract**		
	Q1. Does the paper indicate the study’s design with a commonly used term in the title or the abstract?	Yes No
**Methods**		
	Q2. What is the observational study type: cohort, case-control, or cross-sectional studies?	Cohort study A case-control study Cross-sectional study The study type is not stated in the paper
	Q3. Were settings or locations mentioned in the method?	Yes No
	Q4. Were relevant dates mentioned in the method?	Yes No
	Q5. Were eligibility criteria for selecting participants mentioned in the method?	Yes No
	Q6. Were sources and methods of selection of participants mentioned in the method?	Yes No
	Q7. Were any efforts to address potential sources of bias described in the method or discussion?	Yes No
	Q8. Which program was used for statistical analysis?	SPSS was used for statistical analysis MedCalc was used for statistical analysis SAS was used for statistical analysis STATA was used for statistical analysis R program was used Another program was used for statistical analysis The program for statistical analysis is not specified
**Results**		
	Q9. Were report numbers of individuals at each stage of the study (eg, numbers potentially eligible, examined for eligibility, confirmed eligible, included in the study, completing follow-up, and analyzed) mentioned in the results?	Yes No
	Q10. Was a flowchart used to show the reported numbers of individuals at each stage of the study?	Yes No
	Q11. Were the study participants’ demographic characteristics (eg, age and sex) given in the results?	Yes No
**Discussion**		
	Q12. Does the discussion part summarize key results concerning study objectives?	Yes No
	Q13. Are the limitations of the study discussed in the paper?	Yes No
	Q14. Is the generalizability of the study discussed in the discussion part?	Yes No
**Funding**		
	Q15. Is the funding of the study mentioned in the paper?	Yes No

This list of 15 questions, 2 multiple-choice and 13 yes or no questions, has been prepared by selecting the STROBE checklist items that can be answered definitively and have clear, nonsubjective responses. Question 1, related to title and abstract, examines the LLMs’ ability to identify and understand research designs and terms that are commonly used, evaluating the model’s comprehension of the concise language typically used in titles and abstracts. Questions 2-8, related to methods, cover various aspects of the study’s methodology, from the type of observational study to the statistical analysis programs used. They test the model’s understanding of the detailed and technical language often found in this section. Questions 9-11, related to results, focus on the accuracy and completeness of reported results, such as participant numbers at each study stage and demographic characteristics. These questions gauge the LLMs’ capability to parse and summarize factual data. Questions 12-14, related to the discussion, involve summarizing key results, discussing limitations, and addressing the study’s generalizability. These questions assess the LLMs’ ability to engage with more interpretive and evaluative content, showcasing their understanding of research impacts and contexts. Question 15, related to funding, tests the LLMs’ attentiveness to specific yet crucial details that could influence the interpretation of research findings.

### Development of Novel RAG-Based LLM Web Application

**Figure 2:**
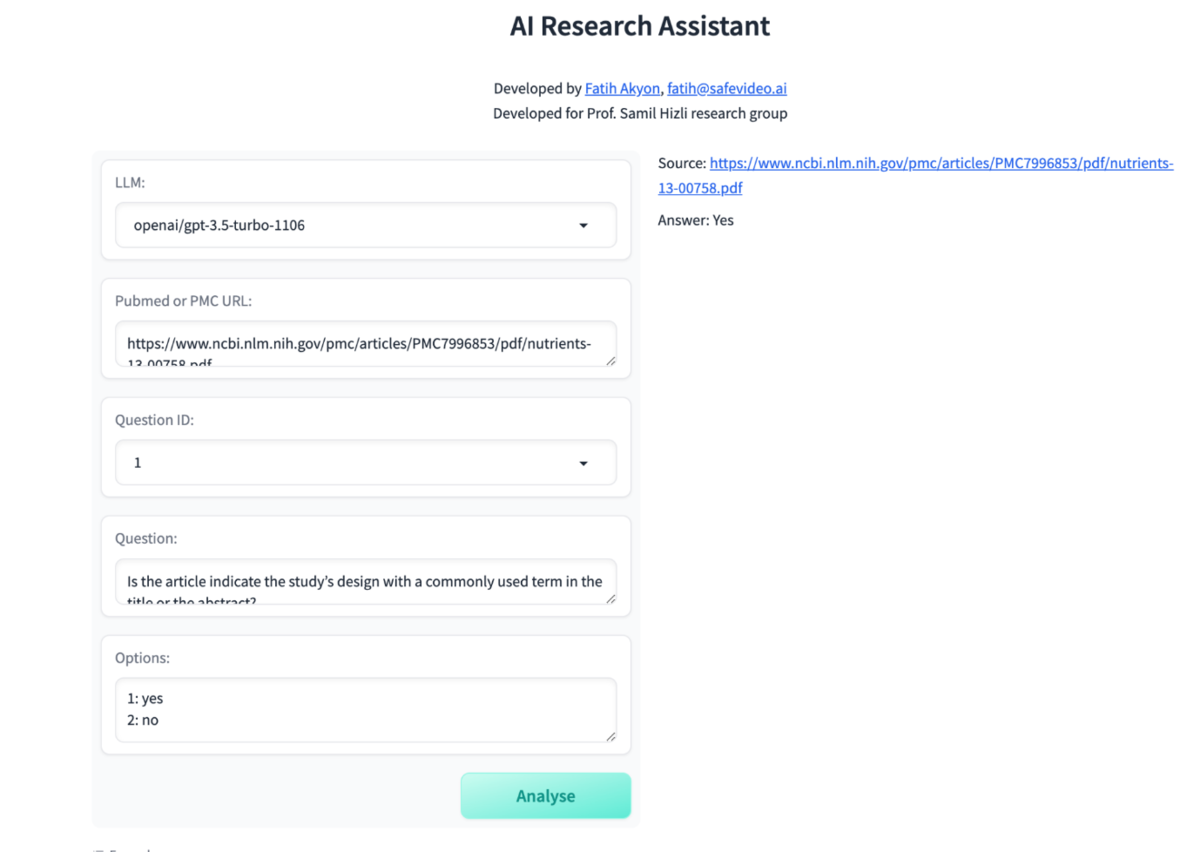
Novel retrieval augmented generation–based large language model web application interface. AI: artificial intelligence.

The methodology incorporated a novel web application specifically designed for this purpose to assess the understanding capabilities of generative AI tools in medical research papers (Figure 2). To mitigate the problem of “artificial hallucinations” inherent to LLMs, this study implemented the RAG method, which involves using a web application to dissect PDF-format medical papers from PubMed into text chunks ready to be processed by various LLMs. This approach guides the LLMs to provide answers grounded in the provided information by supplying them with relevant text chunks retrieved from the target paper.

### Benchmark Pipeline

The benchmark pipeline itself is designed to process PubMed papers of varying lengths and extract relevant information for analysis. This pipeline operates as follows:

- Paper retrieval: We retrieved 39 observational studies from PubMed using the search term “obesity” in the title.
- Text extraction and chunking: Each retrieved PubMed paper was converted to PDF format and then processed through our web application. The application extracts all text content from the paper and divides it into smaller text chunks of manageable size.
- Vector representation: Using the OpenAI text-ada-embedding-002 model, each text chunk was converted into a representation vector. These vectors capture the semantic meaning of the text chunks, allowing for efficient information retrieval.
- Vector database storage: The generated representation vectors were stored in a vector database (LanceDB in our case). This database allows for rapid searching and retrieval of the most relevant text chunks based on a given query.
- Query processing: When a query (question from the STROBE checklist) was posed to an LLM, our pipeline calculated the cosine similarities between the query’s representation vector and the vectors stored in the database. This identified the most relevant text chunks from the paper.
- RAG: The retrieved text chunks, along with the original query, were then combined and presented to the LLM. This approach, known as RAG, ensured that the LLM’s responses were grounded in the specific information present in the paper, mitigating the risk of hallucinations.
- Answer generation and evaluation: The LLM generated an answer to the query based on the provided text chunks. The accuracy of each LLM’s response was then evaluated by comparing it to the benchmark answers provided by a medical professor.

### LLMs

Using this benchmark pipeline, we compared the answers of the generative AI tools, such as GPT-3.5-Turbo-1106 (June 11th version), GPT-4-0613 (November 6th version), GPT-4-1106 (June 11th version), PaLM 2 (chat-bison), Claude v1, and Gemini Pro, with the benchmark in 15 questions for 39 medical research papers (Table 2). In this study, 15 questions selected from the STROBE checklists were posed 10 times each for 39 papers to 6 different LLMs.

**Table 2:** The generative artificial intelligence (AI) tools compared with the benchmark in study.

Generative AI tool	Version	Company	Cutoff date
GPT-3,5-Turbo	November 6, 2023	OpenAI	September 2021
GPT-4-0613	June 13, 2023	OpenAI	September 2021
GPT-4-1106	November 6, 2023	OpenAI	April 2023
Claude v1	Version 1	Anthropic	—^a^
PaLM 2	Chat-bison	Google	—
Gemini Pro	1.0	Google	—

^a^The company does not explicitly state a cutoff date.

Access issues with Claude v1, specifically restrictions on its ability to process certain medical information, resulted in the exclusion of data from 6 papers, limiting the study’s scope to 33 papers. LLMs commonly provide a “knowledge-cutoff” date, indicating the point at which their training data ends and they may not have access to the most up-to-date information. With some LLMs, however, the company does not explicitly state a cutoff date. The explicitly stated cutoff dates are given in Table 2, based on the publicly available information for each LLM.

A chatbot conversation begins when a user enters a query, often called a system prompt. The chatbot responds in natural language within a second, creating an interactive, conversation-like exchange. This is possible because the chatbot understands context. In addition to the RAG method, providing LLMs with well-designed system prompts that guide them to stay relevant to a given document can help generate responses that align with the provided information. We used the following system prompt for all LLMs:

You are an expert medical professor specialized in pediatric gastroenterology hepatology and nutrition, with a detailed understanding of various research methodologies, study types, ethical considerations, and statistical analysis procedures. Your task is to categorize research articles based on information provided in query prompts. There are multiple options for each question, and you must select the most appropriate one based on your expertise and the context of the research article presented in the query.

The language models used in this study rely on statistical models that incorporate random seeds to facilitate the generation of diverse outputs. However, the companies behind these LLMs do not offer a stable way to fix these seeds, meaning that a degree of randomness is inherent in their responses. To further control this randomness, we used the “temperature” parameter within the language models. This parameter allows for adjustment of the level of randomness, with a lower temperature setting generally producing more deterministic outputs. For this study, we opted for a low-temperature parameter setting of 0.1 to minimize the impact of randomness. Despite these efforts, complete elimination of randomness is not possible. To further mitigate its effects and enhance the consistency of our findings, we repeated each question 10 times for the same language model. By analyzing the responses across these 10 repetitions, we could determine the frequency of accurate and consistent answers. This approach helped to identify instances where the LLM’s responses were consistently aligned with the benchmark answers, highlighting areas of strength and consistency in comprehension.

### Statistical Analysis

Each question was repeated 10 times in the same time period to obtain answers from multiple LLMs and ensure the consistency and reliability of responses. Consequently, the responses to the same question were analyzed to determine how many aligned with the benchmark, and the findings were examined. Only the answers that were correct and followed the instructions provided in the question text were considered “correct.” Ambiguous answers, evident mistakes, and responses with an excessive number of candidates were considered incorrect. The data were carefully examined, and the findings were documented and analyzed. Each inquiry and its response formed the basis of the analysis. Various descriptive statistical tests were used to assess the data presented as numbers and percentages. The Shapiro-Wilk test was used to assess the data’s normal distribution. The Kruskal-Wallis and Pearson chi-square tests were used in the statistical analysis. Type I error level was accepted as 5% in the analyses performed using the SPSS (version 29.0; IBM Corp).

### Ethical Considerations

This study only used information that had already been published on the internet. Ethics approval is not required for this study since it did not involve any human or animal research participants. This study did not involve a clinical trial, as it focused on evaluating the capabilities of AI tools in understanding medical papers.

## Results

In this study, 15 questions selected from the STROBE checklists were posed 10 times each for 39 papers to 6 different LLMs. Access issues with Claude v1, specifically restrictions on its ability to process certain medical information, resulted in the exclusion of data from 6 papers, limiting the study’s scope to 33 papers. The percentage of correct answers for each LLM is shown in Table 3, with GPT-3.5-Turbo achieving the highest rate (n=3916, 66.9%), followed by GPT-4-1106 (n=3837, 65.6%), PaLM 2 (n=3632, 62.1%), Claude v1 (n=2887, 58.3%), Gemini Pro (n=2878, 49.2%), and GPT-4-0613 (n=2580, 44.1%).

**Table 3:** The total amounts of correct answers among large language models (LLMs).

LLM	Total questions asked	Correct answers, n (%)
GPT-3.5-Turbo-1106	5850	3916 (66.9)
GPT-4-0613	5850	2580 (44.1)
GPT-4-1106	5850	3837 (65.6)
Claude v1	4950	2887 (58.3)
PaLM 2-chat-bison	5850	3632 (62.1)
Gemini Pro	5850	2878 (49.2)

Each LLM was compared with another LLM that provided a lower percentage of correct answers. Statistical analysis using the Kruskal-Wallis test revealed statistically significant differences between the LLMs (*P*<.001). The lowest correct answer percentage was provided by GPT-4-0613, at 44.1% (n=2580). Gemini Pro yielded 49.2% (n=2878) correct answers, significantly higher than GPT-4-0613 (*P*<.001). Claude v1 yielded 58.3% (n=2887) correct answers, statistically significantly higher than Gemini Pro (*P*<.001). PaLM 2 achieved 62.1% (n=3632) correct answers, significantly higher than Claude v1 (*P*<.001). GPT-4-1106 achieved 65.6% (n=3837) correct answers, significantly higher than PaLM 2 (*P*<.001). The difference between GPT-4-1106 and GPT-3.5-Turbo-1106 was not statistically significant (*P*=.06). Of the 39 papers analyzed, 28 (71.8%) were published before the training data cutoff date for GPT-3.5-Turbo and GPT-4-0613, while all 39 (100%) papers were published before the cutoff date for GPT-4-1106. Explicit cutoff dates for the remaining LLMs (Claude, PaLM 2, and Gemini Pro) were not publicly available and therefore could not be assessed in this study. When all LLMs are collectively considered, the 3 questions receiving the highest percentage of correct answers were question 12 (n=4025, 68.3%), question 13 (n=3695, 62.8%), and question 10 (n=3565, 60.5%). Conversely, the 3 questions with the lowest percentage of correct responses were question 8 (n=1971, 33.5%), question 15 (n=2107, 35.8%), and question 1 (n=2147, 36.5%; Table 4).

**Table 4:** Correct answer percentages of large language models (LLMs) for each question.

Question	Correct answers (across all LLMs), n (%)
Q1	2147 (36.5)
Q2	3061 (52)
Q3	2953 (50.2)
Q4	2713 (46.2)
Q5	3353 (57.1)
Q6	3132 (53.3)
Q7	2530 (43)
Q8	1971 (33.5)
Q9	2288 (38.9)
Q10	3565 (60.5)
Q11	3339 (56.9)
Q12	4025 (68.3)
Q13	3695 (62.8)
Q14	2578 (43.8)
Q15	2107 (35.8)

**Figure 3:**
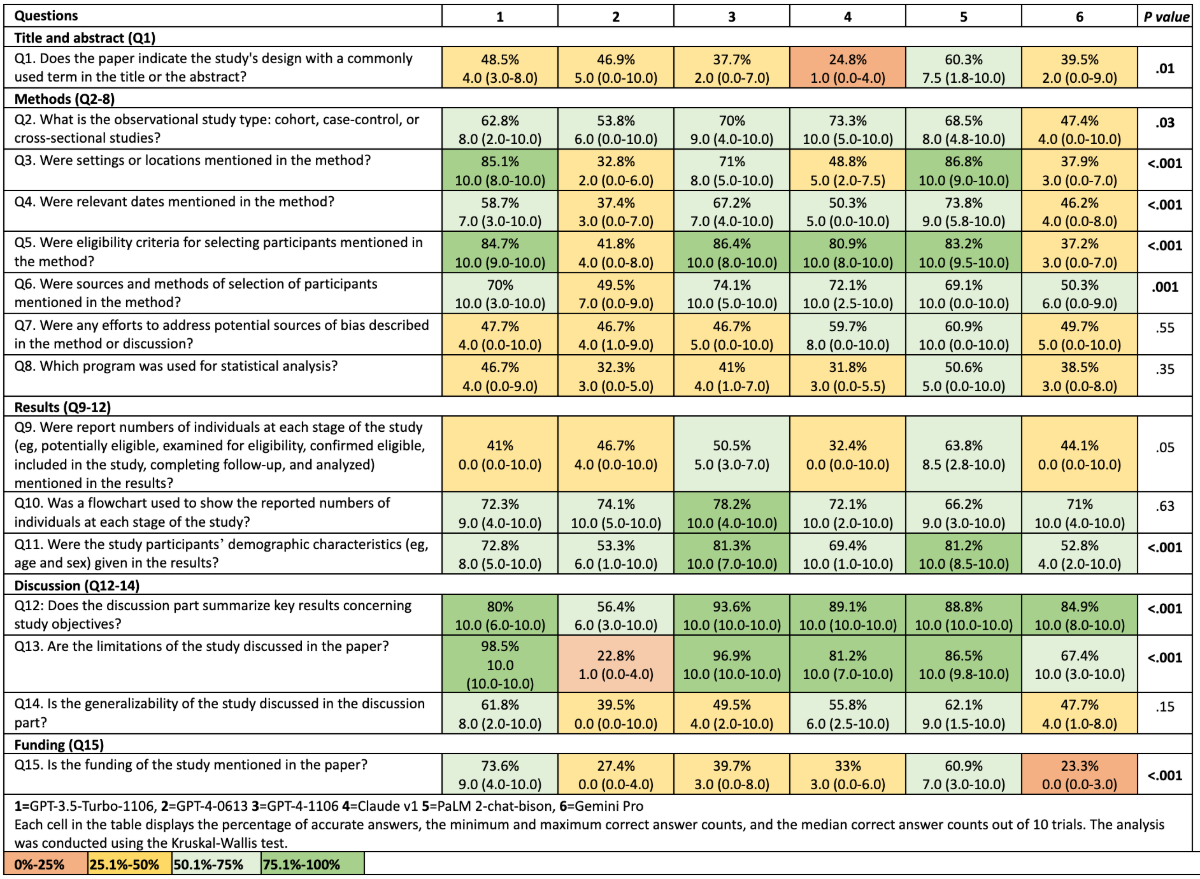
Comparative analysis of correct responses by large language models across 10 iterations for each question.

The percentages of correct answers given by all LLMs for each question are depicted in Figure 3. The median values for questions 7, 8, 9, 10, and 14 were similar across all LLMs, indicating a general consistency in performance for these specific areas of comprehension. However, significant differences were observed in the performance of different LLMs for other questions. The statistical tests used in this analysis were the Kruskal-Wallis test for comparing the medians of multiple groups and the chi-square test for comparing categorical data. For question 1, the fewest correct answers were provided by Claude (n=124, 24.8%) and Gemini Pro (n=197, 39.5%), while the most correct answers were provided by PaLM 2 (n=301, 60.3%; *P*=.01). In question 2, Claude v1 (n=366, 73.3%) achieved the highest median correct answer count (10.0, IQR 5.0-10.0), while Gemini Pro provided the fewest correct answers (n=237, 47.4%; *P*=.03). For question 3, GPT-3.5 (n=425, 85.1%) and PaLM 2 (n=434, 86.8%) had the highest median correct answer counts, while GPT-4-0613 (n=164, 32.8%) and Gemini Pro (n=189, 37.9%) had the lowest (*P*<.001). In the fourth question, PaLM 2 (n=369, 73.8%), GPT-3.5 (n=293, 58.7%), and GPT-4-1106 (n=336, 67.2%) performed best, while GPT-4-0613 (n=187, 37.4%) showed the lowest performance (*P*<.001). For questions 5 and 6, GPT-4-0613 (n=209, 41.8%) and Gemini Pro (n=186, 37.2%) provided fewer correct answers compared to the other LLMs (*P*<.001 and *P*=.001, respectively). In question 11, GPT-4-1106 (n=406, 81.2%), Claude (n=347, 69.4%), and PaLM 2 (n=406, 81.2%) performed well, while Gemini Pro (n=264, 52.8%) had the fewest correct answers (*P*=.001). For questions 12 and 13, all LLMs, except GPT-4-0613, performed well in these areas (*P*<.001). In question 15, GPT-3.5 (n=368, 73.6%) showed the highest number of correct answers (*P*<.001; Multimedia Appendix 1).

## Discussion

### Principal Findings

AI can improve the data analysis and publication process in scientific research while also being used to generate medical papers [16]. Although these fraudulent papers may appear well-crafted, their semantic inaccuracies and errors can be detected by expert readers upon closer examination [11,17]. The impact of LLMs on health care is often discussed in terms of their ability to replace health professionals, but their significant impact on medical and research writing applications and limitations is often overlooked. Therefore, physicians involved in research need to be cautious and verify information when using LLMs. As their reliance can lead to ethical concerns and inaccuracies, the scientific community should be vigilant in ensuring the accuracy and reliability of AI tools by using them as aids rather than replacements, understanding their limitations and biases [10,18]. With millions of papers published annually, AI could generate summaries or recommendations, simplifying the process of gathering evidence and enabling researchers to grasp important aspects of scientific results more efficiently [18]. Moreover, there is limited research focused on assessing the comprehension of academic papers.

This study aimed to evaluate the ability of 6 different LLMs to understand medical research papers using the STROBE checklist. We used a novel benchmark pipeline that processed 39 PubMed papers, posing 15 questions derived from the STROBE checklist to each model. The benchmark was established using the answers provided by an experienced medical professor and validated by an epidemiologist, serving as a reference standard against which the LLMs’ responses were compared. To mitigate the problem of “artificial hallucinations” inherent to LLMs, our study implemented the RAG method, which involves using a web application to dissect PDF-format medical papers into text chunks and present them to the LLMs.

Our findings reveal significant variation in the performance of different LLMs, suggesting that LLMs are capable of understanding medical papers to varying degrees. While newer models like GPT-3.5-Turbo and GPT-4-1106 generally demonstrated better comprehension, GPT-3.5-Turbo outperformed even the more recent GPT-4-0613 in certain areas. This unexpected finding highlights the complexity of LLM performance, indicating that simple assumptions about newer models consistently outperforming older ones may not always hold true. The impact of training data cutoffs on LLM performance is a critical consideration in evaluating their ability to understand medical research [19]. While we were able to obtain explicitly stated cutoff dates for GPT-3.5-Turbo, GPT-4-1106, and GPT-4-0613, this information was not readily available for the remaining models. This lack of transparency regarding training data limits our ability to definitively assess the impact of knowledge cutoffs on model performance. The observation that all 39 papers were published before the cutoff date for GPT-4-1106, while only 28 papers were published before the cutoff date for GPT-3.5-Turbo and GPT-4-0613, suggests that the knowledge cutoff may play a role in the observed performance differences. GPT-4-1106, with a more recent knowledge cutoff, has access to a larger data set, potentially including information from more recently published research. This could contribute to its generally better performance compared to GPT-3.5-Turbo. However, it is important to note that GPT-3.5-Turbo still outperformed GPT-4-0613 in specific areas, even with a similar knowledge cutoff. This suggests that factors beyond training data (eg, the number of layers, the type of attention mechanism, or the use of transformers) and compression techniques (eg, quantization, pruning, or knowledge distillation) may also play a significant role in LLM performance. Future research should prioritize transparency regarding training data cutoffs and aim to standardize how LLMs communicate these crucial details to users.

This study evaluated the performance of various LLMs in accurately answering specific questions related to different sections of a scholarly paper: title and abstract, methods, results, discussion, and funding. The results shed light on which LLMs excel in specific areas of comprehension and information retrieval from academic texts. PaLM 2 (n=219, 60.3%) showed superior performance in question 1, identifying the study design from the title or abstract, suggesting enhanced capability in understanding and identifying specific terminologies. Claude (n=82, 24.8%) and Gemini Pro (n=154, 39.5%), however, lagged, indicating a potential area for improvement in terminology recognition and interpretation. Claude v1 (n=242, 73.3%) and PaLM 2 (n=295, 86.8%) exhibited strong capabilities in identifying methodological details, such as observational study types and settings or locations (questions 2-8). This suggests a robust understanding of complex methodological descriptions and the ability to distinguish between different study frameworks. For questions regarding the results section (questions 9-11), it is evident that models like GPT-4-1106 (n=317, 81.3%), Claude (n=229, 69.4%), and PaLM 2 (n=276, 81.2%) showed superior performance in providing correct answers related to the study participants’ demographic characteristics and the use of flowcharts. All LLMs except for GPT4-0613 (n=89, 22.8%) exhibited remarkable competence in summarizing key results, discussing limitations, and addressing the generalizability of the study (questions 12-14), which are critical aspects of the discussion section. GPT-3.5 (n=287, 73.6%) particularly excelled in identifying the mention of funding (question 15), indicating a nuanced understanding of acknowledgments and funding disclosures often nuanced and embedded toward the end of papers. Across the array of tested questions, both GPT-3.5 and PaLM 2 exhibit remarkable strengths in understanding and analyzing scholarly papers, with PaLM 2 generally showing a slight edge in versatility, especially in interpreting methodological details and study design. GPT-3.5, while strong in discussing study limitations, generalized findings, and funding details, indicates that improvements can be made in extracting complex methodological information. We observed that different models excelled in different areas, indicating that no single LLM currently demonstrates universal dominance in medical paper understanding. This suggests that factors like training data, model architecture, and question complexity influence performance, and further research is needed to understand the specific contributions of each factor.

### Comparison to Prior Work

LLMs can be directly questioned and can generate answers from their own memory [11]. This has been extensively studied in many medical papers*.* According to a study, ChatGPT, an LLM, was evaluated on the United States Medical Licensing Examination. The results showed that GPT performed at or near the passing threshold for examinations without any specialized training, demonstrating a high level of concordance and insight in its explanations. These findings suggest that LLMs have the potential to aid in medical education and potentially assist with clinical decision-making [5,20]. Another study aimed to evaluate the knowledge level of GPT in medical education by assessing its performance in a multiple-choice question examination and its potential impact on the medical examination system. The results indicated that GPT achieved a satisfactory score in both basic and clinical medical sciences, highlighting its potential as an educational tool for medical students and faculties [21]. Furthermore, GPT offers information and aids health care professionals in diagnosing patients by analyzing symptoms and suggesting appropriate tests or treatments. However, advancements are required to ensure AI’s interpretability and practical implementation in clinical settings [8]. The study conducted in October 2023 explored the diagnostic capabilities of GPT-4V, an AI model, in complex clinical scenarios involving medical imaging and textual patient data. Results showed that GPT-4V had the highest diagnostic accuracy when provided with multimodal inputs, aligning with confirmed diagnoses in 80.6% of cases [22]. In another study, GPT-4 was instructed to address the case with multiple-choice questions followed by an unedited clinical case report that evaluated the effectiveness of the newly developed AI model GPT-4 in solving complex medical case challenges. GPT-4 correctly diagnosed 57% of the cases, outperforming 99.98% of human readers who were also tasked with the same challenge [23]. These studies highlight the potential of multimodal AI models like GPT-4 in clinical diagnostics, but further investigation is needed to uncover biases and limitations due to the model’s proprietary training data and architecture.

There are few studies in which LLMs are directly questioned, and their capacities to produce answers from their own memories are compared with each other and expert clinicians. In a study, GPT-3.5 and GPT-4 were compared to orthopedic residents in their performance on the American Board of Orthopaedic Surgery written examination, with residents scoring higher overall, and a subgroup analysis revealed that GPT-3.5 and GPT-4 outperformed residents in answering text-only questions, while residents scored higher in image interpretation questions. GPT-4 scored higher than GPT-3.5 [24]. A study aimed to evaluate and compare the recommendations provided by GPT-3 and GPT-4 with those of primary care physicians for the management of depressive episodes. The results showed that both GPT-3.5 and GPT-4 largely aligned with accepted guidelines for treating mild and severe depression while demonstrating a lack of gender or socioeconomic biases observed among primary care physicians. However, further research is needed to refine the AI recommendations for severe cases and address potential ethical concerns and risks associated with their use in clinical decision-making [25]. Another study assessed the accuracy and comprehensiveness of health information regarding urinary incontinence generated by various LLMs. By inputting selected questions into GPT-3.5, GPT-4, and Gemini, the researchers found that GPT-4 performed the best in terms of accuracy and comprehensiveness, surpassing GPT-3.5 and Gemini [26]. According to a study that evaluates the performance of 2 GPT models (GPT-3.5 and GPT-4) and human professionals in answering ophthalmology questions from the StatPearls question bank, GPT-4 outperformed both GPT-3.5 and human professionals on most ophthalmology questions, showing significant performance improvements and emphasizing the potential of advanced AI technology in the field of ophthalmology [27]. Some studies showed that GPT-4 is more proficient, as evidenced by scoring higher than GPT-3.5 in both multiple-choice dermatology examinations and non–multiple-choice cardiology heart failure questions from various sources and outperforming GPT-3.5 and Flan-PaLM 540B on medical competency assessments and benchmark data sets [28-30]. In a study conducted on the proficiency of various open-source and proprietary LLMs in the context of nephrology multiple-choice test-taking ability, it was found that their performance on 858 nephSAP questions ranged from 17.1% to 30.6%, with Claude 2 at 54.4% accuracy and GPT-4 at 73.3%, highlighting the potential for adaptation in medical training and patient care scenarios [31]. To our knowledge, this is the first study to assess the performance of evaluating medical papers and understanding the capabilities of different LLMs. The findings reveal that the performance of LLMs varies across different questions, with some LLMs showing superior understanding and answer accuracy in certain areas. Comparative analysis across different LLMs showcases a gradient of capabilities. The results revealed a hierarchical performance ranking as follows: GPT-4-1106 equals GPT-3.5-Turbo, which is superior to PaLM 2, followed by Claude v1, then Gemini Pro, and finally, GPT-4-0613. Similar to the literature review, GPT-4-1106 and GPT-3.5 showed improved accuracy and understanding compared to other LLMs. This mirrors wider literature trends, indicating LLMs’ rapid evolution and increasing sophistication in handling complex medical queries. Notably, GPT-3.5-Turbo showed better performance than GPT-4-0613, which may be counterintuitive, considering the tendency to assume newer iterations naturally perform better. This anomaly in performance between newer and older versions can be attributed to the application of compression techniques in developing new models to reduce computational costs. While these advancements make deploying LLMs more cost-effective and thus accessible, they can inadvertently compromise the performance of LLMs. The notable absence of responses from PaLM in certain instances, actually stemming from Google’s policy to restrict the use of its medical information, presents an intriguing case within the scope of our discussion. Despite these constraints, PaLM’s demonstrated high performance in other areas is both surprising and promising. This suggests that even when faced with limitations on accessing a vast repository of medical knowledge, PaLM’s underlying architecture and algorithms enable it to make effective use of the information it can access, showcasing the robust potential of LLMs in medical settings even under restricted conditions.

### Strengths and Limitations

While LLMs can be directly questioned and generate answers from their own memory, as demonstrated in numerous studies above, this approach can lead to inaccuracies known as hallucinations. Hallucinations in LLMs have diverse origins, encompassing the entire spectrum of the capability acquisition process, with hallucinations primarily categorized into 3 aspects: training, inference, and data. Architecture flaws, exposure bias, and misalignment issues in both pretraining and alignment phases induce hallucinations. To address this challenge, our study used the RAG method, ensuring that the LLMs’ responses were grounded in factual information retrieved from the target paper. The RAG method intuitively addresses the knowledge gap by conditioning language models on relevant documents retrieved from an external knowledge source [12,32]. RAG provides the LLM with relevant text chunks extracted from the specific paper being analyzed. This ensures that the LLM’s responses are directly supported by the provided information, reducing the risk of hallucination. While a few studies have explored the use of RAG to compare LLMs, like the one demonstrating GPT-4’s improved accuracy with RAG for interpreting oncology guidelines [33], our study is the first to evaluate LLM comprehension of medical research papers using this method. This method conditions LLMs on relevant documents retrieved from an external knowledge source, ensuring their answers are grounded in factual information. The design of system prompts is crucial for LLMs, as it provides context, instructions, and formatting guidelines to ensure the desired output [34]. In this study, it is empirically determined that a foundational system and set of system prompts universally enhanced the response quality across all language models tested. This approach was designed to optimize the comprehension and summarization capabilities of each generative AI tool when processing medical research papers. The specific configuration of system settings and query structures we identified significantly contributed to improving the accuracy and relevance of the models’ answers. These optimized parameters were crucial in achieving a more standardized and reliable evaluation of each model’s ability to understand complex medical texts. While further research is needed to fully understand the effectiveness of RAG across different medical scenarios, our findings demonstrate its potential to enhance the reliability and accuracy of LLMs in medical research comprehension.

This study, while offering valuable insights, is subject to several limitations. The selection of 50 papers focused on obesity, and the use of a specific set of 15 STROBE-derived questions might not fully capture the breadth of medical research. Additionally, the reliance on binary and multiple-choice questions restricts the evaluation of LLMs’ ability to provide nuanced answers. The rapid evolution of LLMs means that the findings might not be applicable to future versions, and potential biases within the training data have not been systematically assessed. Furthermore, the study’s reliance on a single highly experienced medical professor as the benchmark, while evaluating, might limit the generalizability of the findings. A larger panel of experts with diverse areas of specialization might provide a more comprehensive reference standard for evaluating LLM performance. Further investigation with a wider scope and more advanced methodologies is needed to fully understand the potential of LLMs in medical research.

### Future Directions

In conclusion, LLMs show promise for transforming medical research, potentially enhancing research efficiency and evidence-based decision-making. This study demonstrates that LLMs exhibit varying capabilities in understanding medical research papers. While newer models generally demonstrate better comprehension, no single LLM currently excels in all areas. This highlights the need for further research to understand the complex interplay of factors influencing LLM performance. Continued research is crucial to address these limitations and ensure the safe and effective integration of LLMs in health care, maximizing their benefits while mitigating risks.

## Appendix

Multimedia Appendix 1 Percentages of correct answers by large language models for each question.

## Abbreviations

AI: artificial intelligence
LLM: large language model
RAG: retrieval augmented generation
STROBE: Strengthening the Reporting of Observational Studies in Epidemiology
