# Supplementary figures and images for: Evaluating the Capabilities of Generative AI Tools in Understanding Medical Papers: Qualitative Study
Source: JMIR Med Inform. 2024 Sep 4;12:e59258. doi: 10.2196/59258 (PMC11411230; doi:10.2196/59258)

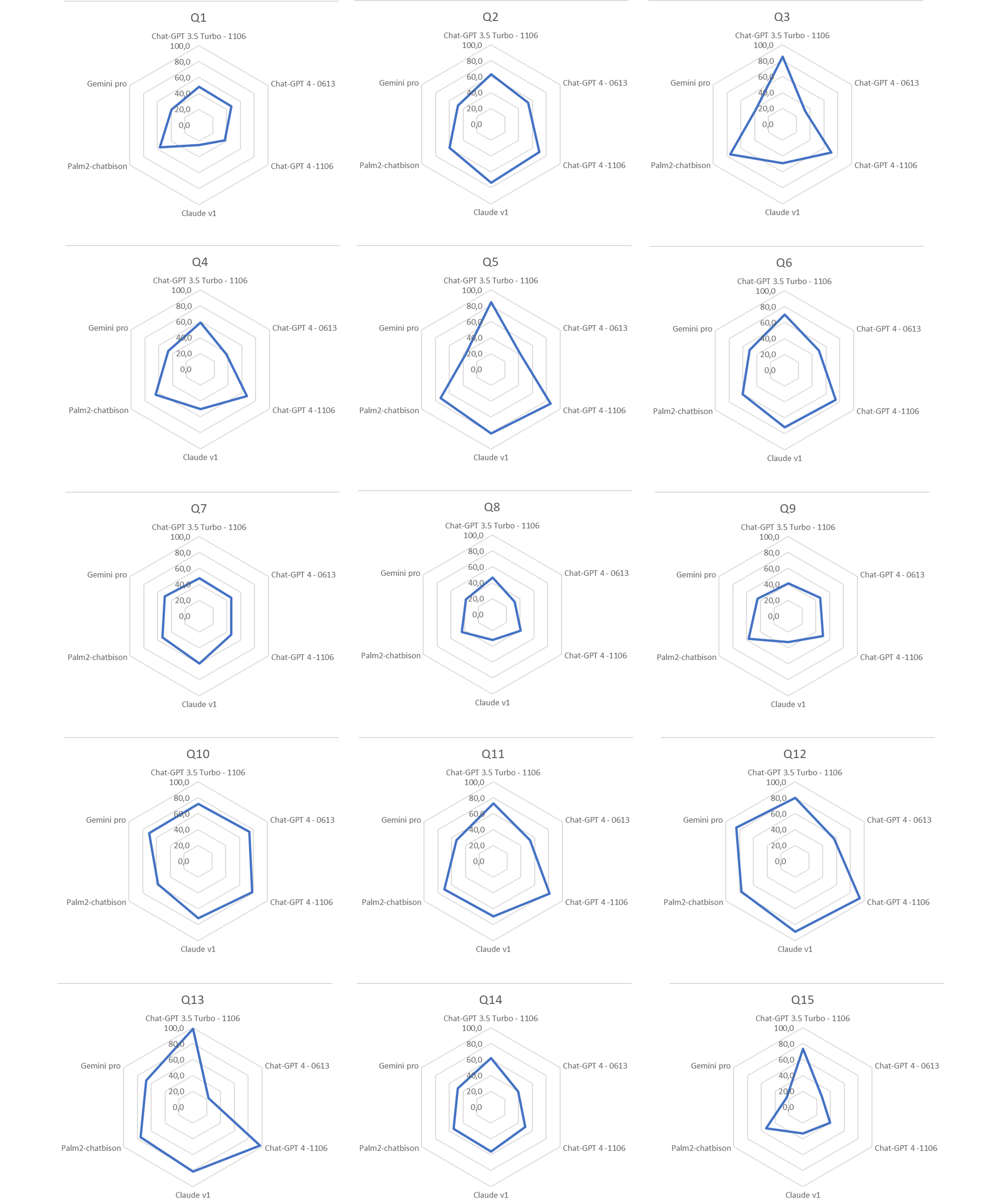

Supplement: Multimedia Appendix 1 [file medinform_v12i1e59258_app1.png]
